# Comparison of a co‐produced mental health service to traditional services: A co‐produced mixed‐methods cross‐sectional study

**DOI:** 10.1111/inm.12681

**Published:** 2019-12-09

**Authors:** Raffaella Pocobello, Tarek el Sehity, Luca Negrogno, Carlo Minervini, Maddalena Guida, Cosimo Venerito

**Affiliations:** ^1^ Istituto di Scienze e Tecnologie della Cognizione Consiglio Nazionale delle Ricerche Rome Italy; ^2^ Faculty of Psychology Sigmund Freud University Vienna Austria; ^3^ Associazione Insieme a noi Modena Italy; ^4^ ASL Brindisi ‐ CSM Brindisi Italy; ^5^ Associazione 180Amici Latiano Brindis Italy

**Keywords:** co‐production, community mental health services, community‐based participatory research, cross‐sectional studies, shared governance, qualitative studies

## Abstract

This study investigates the differences between a co‐produced experimental mental health centre and traditional day centres. For this purpose, we used a collaborative and mixed‐method approach in two complementary studies: (i) a quantitative cross‐sectional study designed to compare users' hospitalization rates and their use of psychiatric medications and (ii) a qualitative study designed to explore and document the experienced differences between co‐produced and traditional services. In the quantitative cross‐sectional study, surveys were administered to 37 users of one co‐produced mental health service and to 40 users of traditional mental health services. A negative binomial regression analysis was performed to examine the relationships between predictors and users' hospitalization rates. After adjusting for the potential confounders, users of the co‐produced centre reported a 63.2% reduced rate of hospitalizations compared with users of traditional mental health services (*P* = 0.002). Furthermore, 39% of users of the co‐produced centre reported a reduction or even withdrawal from psychiatric medications against 22% of the comparison group (*P* = 0.036). In the qualitative study, six main differences emerged from a thematic analysis of a large user‐led focus group. In the participants' experiences, the co‐produced service focused on (i) parity and respectful relationships, (ii) people's strengths, (iii) freedom, (iv) psychological continuity, (v) social inclusion, and (vi) recovery orientation. Our research provides empirical evidence concerning the ‘preventive aspect’ of co‐produced mental health services. Additionally, new insights into how different stakeholders, particularly users of co‐produced mental health services, experience the differences between co‐produced and traditional mental health services are provided.

## Introduction

Over the past decade, co‐production has emerged as an important and challenging area for recovery‐oriented mental health services and has recently attracted the attention of the scientific community (Gordon & O'Brien 2018). Consistent with the recovery‐oriented approach (Anthony, 1993; Pocobello & el Sehity 2012), co‐produced services emphasize the active and valuable roles of users, family members, and citizens in providing the services needed in the mental health sector. Co‐production has been defined as a ‘theory with a set of values and principles, rather than a model' (Roper *et al*. 2018; p. 1), based on the delivery of ‘an equal and reciprocal relationship between professionals, people using services, their families and their neighbors. Where activities are co‐produced in this way, both services and neighborhoods become far more effective agents of change' (Boyle & Harris 2009; p. 11).

Guidelines on co‐production have been developed based on different implementation experiences (Carr *et al*. 2019). Cost‐effectiveness, health benefits, prevention, improvement of practical skills, and social capital are considered the strengths of co‐production experiences (Needham & Carr 2009). Moreover, the first research findings on co‐produced projects are promising. For example, based on her personal experience, Susan Fairlie (2015) argues that co‐production principles and practices empower the people involved and create better services. In particular, the correlation between staff engagement and patient outcomes provides evidence for the incorporation of co‐production approaches into the organizational and operative efforts of mental health services. A literature review on co‐production (Slay & Stephens 2013) reveals an association between co‐production and positive outcomes related to well‐being, social connectedness, stigma, inclusion, personal competencies, and skills, with a positive social return on investment. Qualitative studies have reported that co‐produced initiatives in mental health care, such as recovery colleges, exert a positive impact on users and promote professional change (Chiaf *et al*. 2016; Cleary *et al*. 2018; Meddings *et al*. 2015; Newman‐Taylor *et al*. 2016; Zabel *et al*. 2016).

Despite these findings and positive expectations, direct evidence for the effectiveness of co‐production in mental health care is still lacking (Clark, 2015). As described in the review by Slay and Stephens (2013), the number of articles is still relatively small and the studies are mainly based only on qualitative accounts, with a lack of research assessing different forms of interventions using quantitative mental health or clinical scales. Moreover, their review notes: ‘a number of studies indicated that their project was preventing more acute needs arising by filling a gap in existing service provision that provides support for people prior to reaching the crisis point. Though the preventative aspect was fairly common, it had not been measured or captured in a consistent way in any of the studies'. (Slay & Stephens 2013; p. 9) Furthermore, a better understanding of the contributions of caregivers to the development of co‐produced services is needed (Bradley, 2015).

In an effort to address these gaps, researchers have recommended implementing ‘mechanisms of continuous monitoring and evaluation of the effectiveness of the co‐production process by collaboratively identifying meaningful outputs for both service and community co‐providers. All partners should be involved in collecting and reflecting on evidence of this joint effort'. (Hatzidimitriadou *et al*. 2012, p. 48). Based on these recommendations, the present study aimed to evaluate an experimental co‐produced centre located in the South of Italy – the Marco Cavallo Center (MCC) – in a co‐produced manner, focusing on the main differences from traditional mental health services.

## Background

### A co‐produced mental health centre in the south of Italy – the MCC

The MCC is a centre co‐produced by the public mental health services and a citizen association with many members with lived experiences of mental health issues and their family members. Opened in 2008 by the initiative of three mental health professionals and a group of volunteers (now the association ‘180amici’), the centre was officially recognized by the Apulia region as an experimental centre of co‐production in 2012.

Consistent with Franco Basaglia's legacy (Basaglia, 2000; Foot, 2014), the centre adopted the assembly as its method for the management of its activities at its inception. These assemblies have not only organizational but also therapeutic and political importance, due to the mutual support and resources emerging from them. An open meeting occurs twice weekly, where users, professionals, family members, and citizens discuss together relevant issues and the management of the centre. The assembly is the place where all activities are decided and planned and established working groups are responsible for its implementation. In addition to regular maintenance activities, such as daily shopping, cooking, and cleaning, peer support groups, cultural, and sports activities in and outside the centre are decided and organized.

### Co‐producing the evaluation project

In 2015, the association ‘180amici’ asked the Italian National Research Council to evaluate the MCC using a collaborative approach (Pocobello, 2011). This approach has been developed based on the social‐cognitive theory of value proposed by Miceli and Castelfranchi (1989), in which an evaluation intrinsically depends on the specific interests and goals of involved agents. In a multi‐agent system, such as the MCC, the stakeholders involved have different perspectives, expectations, and evaluations of the centre. These differences between stakeholders ‘*need to be brought to the surface, clarified, articulated and understood before professionals and consumers can expect to work harmoniously'* (McAllister & Walsh 2004, p. 28). Collaborative evaluation is particularly useful to promote a process that increases consciousness, the sharing of ideas, discussions, and negotiations, and may become an essential instrument of co‐responsibility, learning, and cooperation (Van Der Meer & Edelenbos 2006). Furthermore, participatory research in the field of mental health is assumed to reduce the asymmetry in power relations between the researcher and researched (Kara, 2017), thus increasing the quality of studies and providing a necessary complement to mainstream research (Rose, 2003).

A one‐day assembly was organized in December 2015 to discuss the evaluation project. The PI (RP) proposed to use the collaborative evaluation design, presenting the argument outlined above. MCC participants (users, family members, and professionals, a total of ~40 participants) welcomed this methodology and considered it to be consistent with the values and principles of the centre. As one of the users noted, a collaborative evaluation would avoid the sensation of being a ‘laboratory rat [Ital. Cavia]’, which was the feeling experienced when enrolled in previous studies. All participants in the MCC shared ideas about the potential objectives of this research. The sharing process was facilitated by the fact that the meeting was arranged in a large circle, the usual format for MCC assemblies. Additionally, the level of involvement of MCC participants during the research process was determined during the first research assembly, including participation in research planning, data collection, transcription, and imputation processes, as well as the interpretation and dissemination of results.

### Ethics

This study was approved by the Ethics Committee of the Institute of Cognitive Sciences and Technologies of the National Research Council in Rome.

### Design

We used a mixed‐method approach: a qualitative study designed to explore participants' experiences and perspectives about the MCC and a cross‐sectional study with a comparison group intended to assess the quantitative differences between MCC and traditional services.

## Quantitative Study

The study is described according to the STROBE Statement checklist (von Elm, *et al*. 2007) for cross‐sectional studies.

### Objectives

The specific objective of the quantitative study was to compare the co‐produced MCC with traditional day centres in the dimensions that are most meaningful to MCC participants. MCC users proposed hospitalization as the primary measure of the cross‐sectional study. Similar to other narratives, hospitalization represents a traumatic event (Wyder *et al*. 2018) that is often associated with physical restraint and the worsening of the mental health condition. Furthermore, MCC users proposed to assess a reduction in the use of psychiatric medications as another quantitative measure. According to their experiences, medication use must be reduced for users to be actively involved in the co‐production process of the centre. Their formulated hypotheses are listed below.

H1: Users of the co‐produced MCC are less likely to be hospitalized than users of a traditional day centre;

H2: The co‐produced mental health service reduces the use of psychiatric medications.

### Methods

#### Study design

A cross‐sectional study was performed.

#### Setting

The study was performed at the co‐produced MCC of Latiano, a municipality of ~15 000 residents in the province of Brindisi (Apulia, Italy), and three other day centres in the same region in the spring of 2016.

While one day centre was located close to the MCC, the others were located approximately 250 km from the MCC. Due to logistics and limited resources for the study, the data collection process was arranged and coordinated with the directors of the three day centres, and one day on which most users of their centre would be present and participate in the survey was specified. All users present on the day of the survey were eligible to participate in the study (*n* = 40).

#### Variables

Two clinical outcomes of co‐production were established in the hypotheses: reduced hospitalization rates and a reduction in psychiatric medication.

#### Data sources/measurement

We developed a questionnaire based on the input received in the first assembly and the subsequent meetings. The questionnaire was pilot‐tested by users of the three services and discussed in a team meeting including service users and professionals from the co‐produced Marco Cavallo centre, who examined the survey for readability, clarity, and comprehensiveness.

Item 6 (see Table 1) explicitly assessed whether frequent visits to the MCC reduced the number of hospitalizations for users and served as cross‐validation for the reported hospitalization rates (Item 3). Item 7 assessed the reduction in psychiatric medication use.

**Table 1:** Data sources/measurements

Variable of interest	Items (sources of data)	Measure	Statistical assessment
Mental health service usage characteristics	(2) User's type of service at the first contact	Category	Descriptive
	(4) Duration of stay at the current centre	Number of months at the current centre	
	(5) Usual frequency of visits to the centre.	Frequenting the MHC	
Hospitalization Rate	(1) Ln[year of study (2016) – user's year of the first contact with the mental health system]	Offset variable for modelling users' number of hospitalizations.	Negative binomial regression modelling;
	(3) How many times have you been hospitalized for mental health reasons?	Count variable of the number of hospitalizations.	
Users' perceived development of hospitalizations	(6) Since I began frequenting the centre, …	(1) …I have never been hospitalized;	Mantel–Haenszel linear‐by‐linear association; 1‐sided *P*;
		(2) …I have been hospitalized less than in prior periods;	
		(3) …there have been no significant changes in the number of hospitalizations;	
		(4) …I have been hospitalized more than in prior periods;	
Reduction in medication	(7) Since I began frequenting the centre, my psychiatric medication use…	(1) …was suspended;	Mantel–Haenszel linear‐by‐linear association; 1‐sided *P*;
		(2) …was reduced;	
		(3) …has not changed;	
		(4) …was increased;	

#### Participants

We surveyed two groups. The first consisted of users of the co‐produced MCC (*n* = 37). The comparison group (CG: *n* = 40) was composed of users from three traditional day centres in the same region to obtain a comparative group. Day centres in this region have a maximum of ~20 users of their services, and not all users were present at the centres each day.

Males (70%) in the MCC sample were significantly over‐represented compared with 48% in the comparison group; furthermore, the MCC sample had more users with a higher education level, and the source of income differed systematically between groups (see Table 2). All of these factors represented potential confounding variables for our between‐subjects research design.

**Table 2:** Socio‐demographics, mental health service usage, and clinical characteristics

Characteristics	Co‐produced MCC (*n* = 37)	Comparison group (*n* = 40)
Socio‐demographic		
Gender	*χ* ^2^ (1, 75) = 4.10, *P* = 0.043	
Men	**26 (70.3%)**	19 (47.5%)
Women	11 (29.7%)	21 (52.5%)
Age *F*(1, 75) = 2.06, *P* = 0.155	*M* = 42.70; *SD* = 9.10	*M* = 45.95; *SD* = 11.33
Education level	*χ* ^2^ (2, 75) = 15.12, *P* = 0.001	
None (*n* = 2) or obligatory school	12 (32.4%)	**30 (75.0%)**
High School	**18 (48.6%)**	9 (22.5%)
Specialized‐/other (*n* = 2) degrees	**7 (18.9%)**	0 (0.0%)
Income/Employment status	*χ2*(3, 74) = 10.43, *P* = 0.015	
Allowance from family	3 (8.3%)	**8 (21.1%)**
Income from employment	**10 (27.8%)**	1 (2.6%)
Pension	22 (61.1%)	**27 (71.1%)**
No income	1 (2.8%)	2 (5.3%)
Marital status	*χ* ^2^ (2, 76) = 0.19, *P* = 0.906	
Married (living with partner)	7 (18.9%)	9 (23.1%)
Divorced/Separated	2 (5.4%)	2 (5.1%)
Single/Widowed	28 (75.7%)	28 (71.8%)
Mental health usage characteristics		
How many years since your first contact with the mental health system? *t*(67) = 1.31, *P* = 0.194	*M* = 15.03, *SD* = 8.48	*M* = 18.24, *SD* = 11.73
MH service of the first contact:	*χ* ^2^ (3, 75) = 12.69, *P* = 0.005	
Psychiatric wards	5 (13.5%)	**15 (39.5%)**
Day centre	1 (2.7%)	**6 (15.8%)**
Community mental health service	***28 (75.7%)***	15 (39.5%)
Other	3 (8.1%)	2 (5.3%)
How many times have you frequented the Center this year?	*χ* ^2^ (2, 77) = 4.77, *P* = 0.092	
Less than one time per month	1 (2.7%)	0 (0.0%)
A few times per month	2 (5.4%)	1 (2.5%)
A few times per week	5 (13.5%)	1 (2.5%)
Almost every day	29 (78.4%)	38 (95.0%)
Clinical characteristics (CORE‐OM clinical cut‐off scores; Evans *et al*. 2002) Practitioner cut‐off*: χ* ^2^ (1, 75) = 0.24, *P* = 0.624		
Non‐clinical population (1 – 9.9)	13 (36.1%)	12 (30.8%)
Clinical population (≥10)	23 (63.9%)	27 (69.2%)

Boldface indicates significant over‐representations.

#### Mental health assessment

We assessed users' mental health conditions using a routine clinical assessment, CORE‐OM (Evans *et al*. 2002), to control for a potential clinical selection bias at the co‐produced mental health centre. The CORE‐OM is a 34‐item generic measure of psychological distress and draws upon the practitioners' views of the most important aspects of mental health to measure. According to Barkham *et al*. (2010), the clinical cut‐off score for the CORE‐OM scale is 10 points. Overall, one‐third of the sample achieved a score less than the clinical cut‐off of 10 points. The proportion of users with a non‐clinical cut‐off score did not differ significantly between groups (*χ^2^*(75, 1) = 0.240; *P* = 0.624).

#### Mental health service (MHS) usage characteristics

More MCC users (75.7%) visited the community mental health centre compared with 39.5% of users of the comparison group. Relatively more users of the comparison group had their first contact with the MHS via the psychiatric ward (39.5%; MCC: 13.5%) and day centres (15.8%; MCC: 2.7%), generally deviating significantly from an assumed equal distribution: *χ^2^*(75, 3) = 12.69; *P* = 0.005 (see Table 2).

### Statistical methods

Our first research question is related to hospitalization rates, which adopts the form of a non‐negative integer and tends to cluster around the values of 0 and/or 1 with lower frequencies observed at higher values and a positive skew in their distribution. Because we found significant differences in the socio‐demographic‐ and MHS‐usage characteristics of both groups (see Table 2 above), our modelling strategy was designed to control for the effects of potential confounding variables on hospitalization rates. A negative binomial regression analysis was performed to control for associations between our predictor (co‐production) and confounding variables (age, gender, income/employment status, and first contact with the MHS) in the effect on hospitalization rates. Negative binomial regression analysis is generally used to test for the effects of associations between a predictor and confounding variables on a count outcome variable when the variance of the count is greater than its mean. We incorporated an offset variable based on the natural logarithm of the number of years of exposure to the mental health system reported by users (*ln[years in MHS]*).

Our second research question is related to the reduced use of psychiatric medications and was investigated with the Mantel–Haenszel linear‐by linear association to asses our 1‐sided hypothesis a logistic regression analysis to control for associations between our predictor (co‐production) and confounding variables.

Missing data were replaced with the mean value if scores for no more than two items were missing. Data were coded and analysed using IBM SPSS Statistics 24.0.

### Quantitative results

When users were asked whether frequenting the centre affected the frequency of their hospitalizations, 57% said that it had, 15% did not know, and 28% did not notice any effect (no difference between the MCC and CG: *χ^2^*(72, 2) = 2.692; *P* = 0.260).

When users were asked whether they noticed any changes in their hospitalization rates since they started to frequent their centre, 59% (MCC: 72%; CG: 46%) stated that they were never hospitalized since they began to frequent their centre; 16% (MCC: 11%; CG: 22%) were hospitalized less frequently than before; 18% (MCC: 17%; CG: 19%) did not note any difference; and 7% (MCC: 0%; CG: 14%) were hospitalized more than before they visited the centre. We treated the item as a variable with four ordered categories and used the Mantel–Haenszel linear‐by‐linear association to asses our 1‐sided hypothesis that co‐produced services reduce hospitalizations: *χ^2^*(1) = 5.75; 1‐sided *P* = 0.016.

H1: Reduced Hospitalization Rates

**Figure 1:**
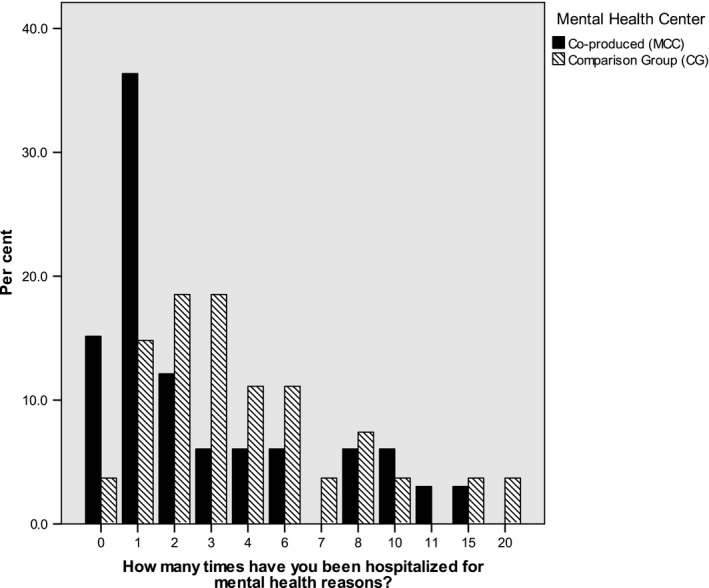
Hospitalization rates per group.

In both samples, the reported number of hospitalizations varied widely between 0 and a maximum of 20 hospitalizations, with a mean of *M*(MCC) = 3.31 (*SD* = 3.89) and *M*(CG) = 4.88 (*SD* = 4.65). Similar to most count data, the reported hospitalization rates were strongly over‐dispersed (DeMaris, 2005), with the variance (17.47) exceeding the mean (3.92) substantially (see Fig. 1); these values deviated significantly from the Poisson distribution (*D*(71) = 3.31, *P *< 0.001).

Consequently, a negative binomial regression analysis was performed to predict users' hospitalization rates based on centres' co‐production versus traditional services. Before adjusting for the confounders observed in Table 2, co‐production was associated with a reduction of 42.7% in the number of hospitalizations reported by users (IRR = 0.57, 95% CI: 0.32–1.02, Wald *χ^2^*(1) = 3.57, *P* = 0.059). After adjusting for the potential confounders listed in Table 2 (gender, age, educational degrees, MHS used at the first contact, and source of income), the co‐produced centre decreased the percentage of hospitalizations by 63.2% (*IRR* = 0.37, 95% CI: 0.19–0.70) compared with the TAU of the CG and increased in statistical significance (Wald *χ^2^*(1) = 9.38, *P* = 0.002).

Additionally, age emerged as a significant predictor (Wald *χ^2^*(1) = 15.75, *P* < 0.001) for hospitalization rates, but should be considered a built‐in artefact of the time required for multiple hospitalization events: every added decade in the age of users (SD = 9.84) predicts about one more hospitalization (IRR = 0.94, 95% CI: 0.91–0.97). Table 3 presents the output of the adjusted negative binomial regression model designed to predict hospitalization rates.

**Table 3:** Negative binomial regression model predicting likelihood of hospitalization

Parameters	B	Std. error	95% Wald CI		Hypothesis test		Sig.	IRR	95% Wald CI for Exp (B)	
			LL	UL	Wald *χ* ^2^	df			LL	UL
(Intercept)	2.90	0.94	1.05	4.74	9.49	1	0.002	18.13	2.87	114.61
Co‐production [MCC = 1]	−1.00	0.33	−1.64	−0.36	9.38	1	0.002	0.37	0.19	0.70
Sex [male = 1]	−0.26	0.29	−.82	0.31	.77	1	0.380	0.78	0.44	1.37
Age (M = 44.89; SD = 9.84)	−0.06	0.02	−0.09	−0.03	15.75	1	0.000	0.94	0.91	0.97
Educational degrees (overall)	—	—	—	—	4.16	2	0.125	—	—	—
None obligatory [1]	−0.89	0.45	−1.77	−0.01	3.96	1	0.046	0.41	0.17	0.99
High school [2]	−0.55	0.42	−1.36	0.27	1.71	1	0.190	0.58	0.26	1.31
MHS service of the first contact (overall)	—	—	—	—	2.78	3	0.427	—	—	—
Psychiatric ward [1]	0.43	0.29	−0.15	1.01	2.14	1	0.144	1.54	0.86	2.73
Day centre [2]	−0.02	0.51	−1.03	0.98	.01	1	0.965	0.98	0.36	2.68
Other [3]	−0.15	0.50	−1.12	0.82	.09	1	0.763	0.86	0.33	2.28
Income situation (overall)	—	—	—	—	.77	3	0.858	—	—	—
No income [0]	−0.69	0.97	−2.58	1.20	.51	1	0.474	0.50	0.08	3.32
Family support [1]	−0.40	0.60	−1.58	0.78	.45	1	0.505	0.67	0.21	2.17
Pension/State [2]	−0.22	0.38	−.96	0.53	.33	1	0.569	0.81	0.38	1.69
(Negative binomial)	0.45	0.14	.24	0.82	—	—	—	—	—	—

Dependent variable: H1 – How many times have you been hospitalized for mental health reasons?

Model: (Intercept), co‐produced centre, educational titles, MH service of the first contact, income situation, Sex: male, Age, offset = exposure Variable: Ln(years in the mental health system); —, em‐dash.

*N* = 55. Cl = confidence interval.

The predicted number of hospitalizations for users (age was fixed as a covariate at *M* = 45 years) amounted in the co‐produced MHS to a mean of 0.18 hospitalizations (SE = 0.63, CI: 0.09–0.36) and in traditional mental health services to a mean of 0.50 hospitalizations (SE = 1.55, CI: 0.27–0.92). This corresponds to a significant reduction in the hospitalization rate of users in the co‐produced MHS of about 64% as compared with the reference group.

H2: Reduced use of Psychiatric Medication

**Figure 2:**
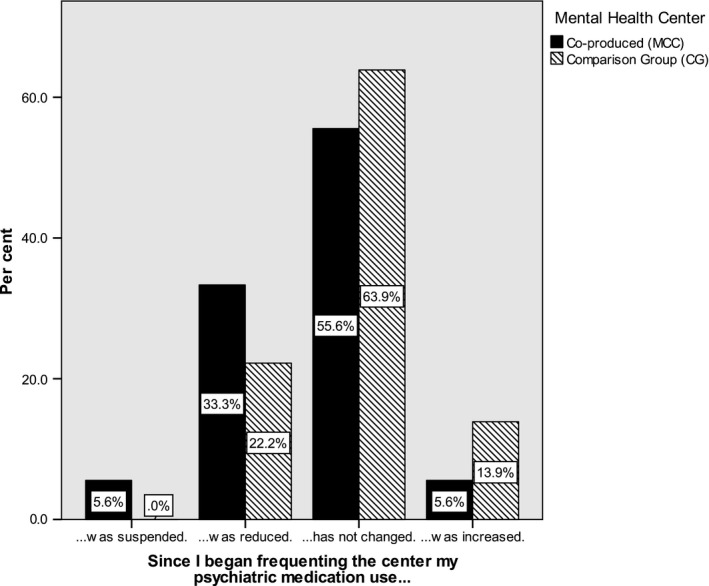
Psychiatric medications per group.

The item responses ‘Since I started to frequent the centre my psychiatric medication (1) … was suspended (2) …was reduced (3) …did not change significantly (4) …were increased' were treated as a variable with four ordered categories. We used the Mantel–Haenszel linear‐by‐linear association to asses our 1‐sided hypothesis that co‐produced services reduce the use of psychiatric medication compared with traditional mental health services: *χ^2^*(1) = 3.85; 1‐sided *P* = 0.036. Overall, 39% of users of the co‐produced MHS reported a reduction or even withdrawal from psychiatric medications against 22% of the comparison group (see Figure 2).

A logistic regression analysis controlling for the confounding effects of the socio‐demographic variables was conducted. Only the MHS‐group variable exerted a significant effect in the expected direction (*B* = −1.63, Wald *χ^2^*(1) = 5.22, *P* = 0.022).

## Qualitative Study

### Objective

Explore the personal experiences of MMC participants (users, professionals, and family members), focusing on the differences between traditional‐ and co‐produced mental health services.

### Description

The study is described according to the COREQ checklist (Tong *et al*. 2007).

### Domain 1: Research team and reflexivity

#### Data collection

A large user‐led focus group was facilitated by a Marco Cavallo user, who proposed to the others the central question of the meeting: ‘*What are the differences you experienced between the Marco Cavallo Center and traditional mental health services if any?'*. Participants sat in a circle and discussed the question.

#### Data analysis

Researchers who analysed the data were not present during the assembly. At the time of the assembly, LN was a doctoral student and RP was a post‐doctoral research fellow. Participants knew that both researchers were interested in the topic of co‐production and were curious about the MCC experience before the commencement of the study.

### Domain 2: Study design

A large user‐led focus group was conducted. MCC participants preferred this approach rather than a series of focus groups, because it was considered more similar to their assembly.

We used purposive homogeneous sampling (Palinkas *et al*. 2015) of people who usually visit the centre. We approached participants collectively and announced the thematic assembly during the routine assembly one month prior to the commencement of the study and repeating it at every weekly meeting.

The sample was composed of 26 participants: 18 MCC users, four young professionals, and four family members. Since face‐to‐face contact or a formal invitation process did not exist, we were unable to determine whether a user refused to participate. The location was the MMC, in the large room usually used for the assembly. All participants knew each other and were actively involved in the assembly.

The single question proposed during the assembly was elaborated by MCC participants together with the researchers in a previous meeting. It was not pilot‐tested nor repeated in other assemblies. The assembly was video‐recorded. Field notes were recorded by RP and LN while watching the video. The duration of the assembly was two and a half hours. Responses were transcribed verbatim and returned to participants for approval.

### Domain 3: Data analysis and findings

We conducted a thematic analysis (Braun & Clarke 2006). Themes were derived from the data using a semantic and constructionist perspective (Patton 2002).

Data analysis was conducted by two researchers in six steps: (i) becoming familiar with the data, watching the video registration, and reading its transcription; (ii) generating initial codes and reporting them using Excel software; (iii) searching for themes; (iv) preparing thematic maps and reviewing themes according to the dual criteria for judging categories proposed by Patton (2002) – internal homogeneity and external heterogeneity; (v) defining and naming themes, assessing the consistency and clarity of major themes; themes were discussed to reach a consensus between coders; and (vi) reporting and validating the research findings together with MCC participants in a dedicated assembly.

### Qualitative results

Despite the large size of the group, the climate of the meeting was peaceful and respectful. Participants had the opportunity to speak at least once and talked about relevant aspects of their experience related to the research question. However, participants were validating one another's claims rather than challenging them. Therefore, participants appeared to contribute ‘to the common communicative ground, which involves referring to what has been said earlier by another participant and gradually constructing a narrative together’ (Lehoux *et al*. 2006; p. 2093).

Following the thematic analysis, six main differences emerged between the MCC and traditional services:

1. Parity/respectful relationship versus Asymmetric relationships;
2. Focus on the strengths of participants versus Focus on the illness;
3. Freedom versus Control;
4. Psychological continuity versus Discontinuity;
5. Social inclusion versus Segregation;
6. Recovery versus Chronicity.

The results are reported in Table 4. Participants' quotations are presented to illustrate the themes, and each excerpt is identified with a code for the role in the centre (user, professional, or family member) and an ID code.

**Table 4:** Identified themes of the thematic analysis

Themes	Data
1 ‐ Parity/ respectful relationship versus Asymmetric relationships	
Feeling respected and treated as a par	User 5 (female): “Above all I can say that here at the Marco Cavallo there is the presence of an association which is quite strong (.) thus, you enter in a service where we are persons ‐ not just educators of mental health, but also simple citizens who are here to do something together (…) rather than finding yourself closed in a room with a specialist, with a psychiatrist or a psychologist seated behind a table who poses questions (.) who exploits and judges us from above…”
	Professional 2 (female): “I was much impressed by the relations with the persons, where you do not instill a sort of asymmetry but parity, where all have a stake and collaborate”
Parity is missing in traditional services	Professional 1 (female): “I have known the mental health center of [place nearby] where I did my internship. The difference you certainly may note at the Marco Cavallo is that at the other center there is a clear distinction between the professional and the user – whereas here at the MCC‐center all these distinctions do not exist”
2 ‐ Focus on people strengths versus Focus on illness	
MCC values each person, focusing on strengths and potentialities	User 1 (male): “What I always say is that here at the MCC you do – here is the Doing. There is much emphasis on challenging the capabilities of each of us, even those hidden. Every day we put ourselves to the test in different situations and bring out our best (.) and for sure we are not abandoned to ourselves, left maybe in a room, watching television or talking about the‐more‐and‐less (.) every day we do something – we can give a lot”
Traditional services focus on illness	Professional 3 (female): “What I certainly have noted is, that the [traditional] mental health centers – in most cases – are ambulatory services. People with mental issues come to the service and ask for a colloquium, psychotherapy, receive a pharmacological therapy (.) so what is valorized most in these services is the aspect of the illness (…) negative aspects of the person. At the MCC instead, the capacities are valorized, the resources of every single person are really put into practice. In the Marco Cavallo, the persons put themselves at stake, mobilize their proper resources and can undertake and practice some activities they never had a chance to do before”
Positive attitude promotes social inclusion and employability	User 11 (male): “This is an important aspect of the center since it does not chronicize but allows the person – with the work – to face the external world, to socialize”
	Professional 4: (female) “The Marco Cavallo must not be considered a destination point because (.) it is clear that it is a sort of trampoline, where people can learn and obtain some capacities which are put to practice in the outside world…”
3 ‐ Freedom versus Control	
Free agency	User 17 (male): “at the Marco Cavallo I find a different ambient (.) I feel freer and do not feel observed”
Feeling obliged and passive in traditional services	User 15 (male): “The Marco Cavallo was and is a place where one liberates his mind because you do something else differently (..) This is for me the Marco Cavallo! There is no waiting line for the therapy; there is not the obsessive cleanliness – this ‘you‐need‐to‐do‐this‐and‐that‐between‐eight‐and‐ten' (.) there is no professional you must give account to, there is no television at a specific hour (.) There are none of these regulations of time which institutions ask for (.) I organize my time by myself (.) I come in the morning with the bus at my free will, because no one here obliges me (…) I have also frequented another day center where there were many laboratories frequented by many people (…) there, we all – almost – have been obliged to do the same exercise, even if we didn't like it (.) here instead I have my arts‐laboratory, where I do what I would like to be able to do…”
Experience of the centre openness in contrast to previous experiences with traditional services	Family member 3 (male): “I remember, when one went to the mental health center, one rang [the bell], entered a room, the door behind us being locked (.) you needed to ring at a different doors to call the nurse who would come out to say: “you need to wait for half an hour, an hour” and then closed the door again. The experience at the mental health center has been the frequentation of a stagnant ambient, the frequentation of interiors, an inward directed psychology and psychiatry. I lamented this. Here I have found, above all, open doors. Even now there are these open doors (.) I can go and direct myself wherever I want to, frequent whatever I want and come at the hours I want to”
4 ‐ Psychological continuity versus discontinuity	
Frustration due to lack of continuity in therapeutic relations at traditional services	User 2 (male): “The [previous] public mental health center has not been very appropriate for me because of the continuous change of psychiatrist and psychologists. In 20 years, I had each year a different one. I believe that I could have stayed better than I am even now if I would have had the same psychiatrist or the same psychologist (.) but overall, I had 20–22. Telling my story all the time from the start has been stressful – it did not make me feel well to remember those things…”
	User 4 (male): “You may get used to talking to get to everything you have inside, with a doctor [but] after a certain period he or she disappeared (.) I needed to restart everything from the start (.) and this – honestly – I did not find right…”
MCC is a place with stable and authentic relations	User 3 (female): “Not having a family I have started one here, in a sane and clean context, where there is no prejudice”
	(Users 4, male): “Here [at the Marco Cavallo], I feel well because you are my friends”
5 ‐ Social inclusion versus Segregation	
Feeling disempowered and out of society in traditional services	User 10 (male): “In the community where I was before for two and a half years, I was annihilated. (.) First, they gave me a lot of psychiatric pharmaceuticals(..) when I came out, I was even not able to take the bus. Let us say, that I went out, had to return home to my mother (.) I forgot almost the life as it was (…) after all these experiences in these communities I had difficulties reinserting myself in society. I was not able to find work, had difficulties. With this day center – the Marco Cavallo – I have found good possibilities to reinsert myself in society”
Experiencing social and work inclusion	Family Member 1 (male): Before we felt isolated from the world, even go out from home was difficult (.) Because, when this situation happens (mental health issues) is like grief in the family, and there is a general pressure. Now, having had the opportunity to be involved in the center, we have understood the difference to our previous life and the actual one (.) It was giving us the possibility to alleviate the family burden and insert our son in a social and even working context, giving him also some economic autonomy
6 ‐ Recovery versus Chronicity	
MCC associated with the concept of recovery – missing in traditional services	Family Member 3 (male): “Here, for the first time, I heard about the concept of recovery which goes beyond the word – but is the result of an organized system in which more persons undertake a journey, something which in ten years of mental health systems, of SPDC (psychiatric ward), of private structures, of psychologists, seems to have been negated (.) all told me that I would need to resign, that it would be definitive and that one would need to organize accordingly. Here at the Marco Cavallo, instead, I feel that I can heal (.) in a couple of years the journey has been one of cure, that of my wife and that of my own (.) because every problem is dealt with from a holistic perspective, more global (.) here there has been the possibility to take on the journey to grow together”
Take on responsibilities and regain self‐confidence	Users 17 (male): “Before I believed that I could rely only on medication, quite heavy medication, on medical controls but I was unable to win my fears (.) My illness started in the '90ies and was quite severe so you can imagine how many medications I have taken (.) In this journey, we are doing here (at MCC) we start wishing to improve our mental health (.) not only based on medication but with the activities we are doing in the center, and, overall, with the fact that we are taking responsibilities, being more self‐confident… I never experienced something like this before”

## Discussion

The present study investigated the differences between a co‐produced mental health centre and traditional day centres using a collaborative research design and a mixed‐method approach. Two independent but complementary studies were performed in the form of one cross‐sectional quantitative study and one qualitative study.

As recommended in previous studies on co‐production (Hatzidimitriadou *et al*. 2012; Roper *et al*. 2018), our cross‐sectional study assessed the main effects and outcomes of co‐produced mental health services identified as purposeful by MCC stakeholders (users, professionals, and family members). We investigated (H1) the reduction in hospitalizations for an acute mental health crisis and (H2) the reduced use of medications. According to a previous study, involvement in co‐produced mental health services potentially prevents needs for acute treatment, since these services provide support before the crisis point, although this ‘preventive aspect’ was never measured consistently (Slay & Stephens 2013). Our study addressed this gap by providing the first evidence for the ‘preventive aspect' related to co‐produced mental health services. MCC users reported having experienced less than half of hospital admissions than did users of the comparison group: The co‐produced Mental Health Center (MHC) decreased the percentage of hospitalizations by 63% as compared to traditional mental health services. Further, 39% of MCC users reported a reduction in or even withdrawal from psychiatric medications against 22% of the comparison group.

Based on these findings, the involvement in a co‐produced service might indeed exert protective effects. This protective factor is potentially related to the accessibility of a social network, peer support, sense of belonging, health promotion, and social inclusion, promoted by the MCC. In an ethnographic study of the MCC (Negrogno, 2017), MCC stakeholders claimed that the protective effect of the centre is related to their commitment to the co‐production process, leading to the use of human and economic resources. According to our reported findings of users' reduced hospitalizations rates, the reduction in medication use, and the substitution of disability pensions with contractual employment due to their work inclusion in the centre, we obtained further evidence that co‐produced services may indeed represent a cost‐effective and sustainable approach for the future of public health services.

Furthermore, our qualitative study provided an understanding of the differences in the views and experiences of users between co‐produced and traditional mental health services. According to the narratives of MCC participants, the centre adheres to the co‐production values identified by Cahn (2000): recognizing peoples' assets, valuing work differently, promoting reciprocity, and building a social network. Similar to the study by Mayer and McKenzie (2017) on the psychological impact of co‐production, MCC users emphasized the possibility of exercising control and perceiving their feelings as being valued and treated with respect as the central themes related to the experience of co‐produced services. In study 2, these themes emerged in stark contrast to their accounts of their experiences with traditional services, which users and professionals related to a lack of freedom, asymmetrical relationships, and a focus on the illness. Consistent with the findings from the study by Haskell *et al*. (2016), users complained about needing to repeat their stories in traditional services due to the psychological discontinuity of care. In contrast, users reported that the MCC provided them with a community of support similar to the assistance provided by a family: stable, authentic, and a place where they feel accepted.

Moreover, study 2 improved our understanding of family members' perspectives about co‐production, which hitherto has been identified as a point of weakness in the literature (Bradley, 2015). In all of the family members' narratives, the MCC was associated with the discovery of the concepts of recovery and hope, which was missing in traditional services. Consistent with the results of other studies (Haskell *et al*. 2016), carers appreciated the inclusiveness and the holistic approach of the centre.

The collaborative design we chose for our study challenged conventional methodological expectations about recruitment and data collection. The same challenges were already reported by Lambert and Carr (2018), who conducted a large focus group with 17 women with physical and mental health needs who were involved in co‐produced research. Nevertheless, we are convinced that this approach provided several benefits to the research. Benefits include the choice of outcomes that are meaningful to participants (Banfield *et al*. 2018, 2012), and a better analysis and interpretation of data due to the inclusion of service users' perspectives (Faulkner & Thomas 2002). The issue of the power imbalance between the researcher and service users is considered a main critical factor in the literature (Kara 2013; Rose 2003; Wallcraft *et al*. 2009) and has been mitigated in our study by the use of the assembly as the main place to make decisions. The assembly represents the core method of the centre, MCC participants always represented the majority, and researchers were challenged by the assembly to avoid the use of jargon and to share knowledge and power, which is critical for the implementation of co‐production (Stomski & Morrison 2017). Moreover, the assembly facilitated the process of systematically considering stakeholders' views, including family members, which are rarely efficiently involved in collaborative research (Kara 2017).

Finally, the participatory research process promoted a sense of belonging and even the proper pride (Miceli *et al*. 2017) of having contributed actively to the research, as shared in the assembly dedicated to the interpretation of the results.

## Limitations and Future Research

Several limitations are worth noting. First, because of its observational and descriptive nature based on a single measurement, the cross‐sectional research design is limited in its ability to identify causal relations. Despite this limitation, our study has provided valuable information to direct further studies designed to establish whether causal relations between co‐production, ‘preventive’, and ‘protective’ mental health aspects are indeed present. Therefore, we strongly recommend longitudinal studies in the future.

Second, the findings based on the characteristics of our sample must be generalized with caution since the sample was small and restricted to users of the same Italian region. A larger multi‐centre study would provide invaluable information about transferability and the impact of the co‐production in various contexts.

Third, we employed only self‐reported measures in the cross‐sectional study. Other sources of information, particularly medical records, might reveal additional or even different effects to the results documented in this study. We collected the data for the qualitative study from a large focus group at the MCC, where participants knew each other, which may have prevented the sharing of critical issues concerning the centre and could partially explain the ‘better–worse’ dichotomy observed in the comparison between MCC and traditional services. In‐depth interviews might have provided better information about critical issues related to one's involvement in co‐produced mental health services. Additionally, the large size of the group limited the possibility of debate.

Finally, we acknowledge the possible bias related to the collaborative approach to recruitment adopted for the MCC. The use of a similar collaborative approach with the control group would have strengthened our study.

## Relevance to Clinical Practice

In a context where co‐production is expanding, this study documents the clinical benefits of a co‐produced service through quantitative data and narratives, encouraging the use of co‐production in mental health practice. According to our quantitative results, involvement in a co‐produced service is related to a decrease of 63% in hospital admissions; 39% of users of the co‐produced MHC report reduction in psychiatric medications. It is worth noting that users chose both these indicators for their relevance. Moreover, according to our qualitative study, users of the co‐produced MHC reported preferring to being involved in co‐production compared with their previous experience of traditional services. Similar to previous research (Horgan *et al*. 2018), participants noted the need for a shift in the language to overcome the distinction between professionals and service users during the participatory process.

## Conclusions

The present study provides the first compelling evidence of the ‘preventive aspect’ associated with co‐produced mental health services. The study provides insights into stakeholders' views and experiences of the differences between co‐produced and traditional services. These findings expand and are consistent with previous research highlighting the benefits of co‐production (Mayer & McKenzie 2017; Needham & Carr 2009; Slay & Stephens 2013). Additional studies are needed to determine the causal relations between benefits and co‐production, assess cost‐effectiveness, and investigate processes and challenges in the implementation, delivery, and outcomes of co‐produced mental health services. Since co‐production challenges conventional methodology, further research and guidelines are needed to address its implications for mental health research.

## Acknowledgements

This research was supported by the Association 180Amici and the Foundation for Excellence in Mental Health Care. We thank Gina Nikkel, Bob Nikkel and Maria Miceli for their input on earlier drafts of this paper, Cristriano Castelfranchi for his mentorship in this research and our colleagues from Association 180Amici who provided their insights and expertise that greatly contributed to the research, although they may not agree with all of the interpretations/conclusions of this paper. We also thank two reviewers whose suggestions helped improve and clarify this manuscript significantly.
